# A Child's Reproof

**Published:** 1889-03-09

**Authors:** 


### A CHILD'S REPROOF.

Miss Margaret Watson writes: I shall be very glad if you will kindly insert the enclosed poem in The Hospital, provided you think it worthy of space. The composer is an invalid child who has been a constant reader of your very interesting and useful paper from its first issue. Being myself a trained nurse (of St. John's House) as well as a lover of children, I trust you will consider the cause an apology for trespassing:

### March On.

March on! ye say, March on!
Hear the world's loud trumpet-call,
Charge! every man to the front,
Prepared to win or fall.

March on, ah yes, march on:
Ye say, "We are Christians all,"
Yet ye give no thought to the helpless ones
Who thick by the roadside fall.

Do ye never, oh children of mammon,
Hear the children's mournful cry?
Ah, yes; yet still never heeding,
Ye leave them alone to die.

Always thus. March on, march on!
Still ye wave your banners and say,
"Homage and wealth await us,"
And we will not halt or stay.

Stop, stop, and listen a moment;
Ye are leaving far behind
All that is loved and honoured
By the noblest of mankind.

Hasten on, once more hasten on,
Not the broad, but the narrow way,
For the goal is worthy the struggle—
Try and gain it without delay.

—*Leon.*

